# Complete Genome Sequence of *Salmonella enterica* Serovar Typhimurium Myophage Mushroom

**DOI:** 10.1128/genomeA.00154-15

**Published:** 2015-04-09

**Authors:** Tamra N. Tolen, Yicheng Xie, Adriana C. Hernandez, Gabriel F. Kuty Everett

**Affiliations:** Center for Phage Technology, Texas A&M University, College Station, Texas

## Abstract

*Salmonella enterica* serovar Typhimurium (*S*. Typhimurium) is a leading cause of foodborne illness worldwide. Over the past two decades, strains resistant to antibiotics have begun to emerge, highlighting the need for alternative treatment strategies such as bacteriophage therapy. Here, we present the complete genome of Mushroom, an *S*. Typhimurium myophage.

## GENOME ANNOUNCEMENT

*Salmonella enterica* serovar Typhimurium (*S*. Typhimurium) is a leading cause of gastroenteritis in humans worldwide. With growing data that shows increasing drug resistance of *S*. Typhimurium, other alternatives, like bacteriophage therapy, are needed (1, 2). Future application of phage therapy in the United States will most certainly require detailed knowledge of the phages utilized (3). Hence, we present the complete genome of *S*. Typhimurium myophage Mushroom. Mushroom is a component of IntestiPhage (developed by the George Eliava Institute of Bacteriophages, Microbiology and Virology, Tbilisi, Georgia), a cocktail of 23 phages active against several enterobacteria strains (4).

Bacteriophage Mushroom was isolated from IntestiPhage (Lot #M2-401). DNA was sequenced in an Illumina MiSeq 250-bp paired-end run with a 550-bp insert library at the Genomic Sequencing and Analysis Facility at the University of Texas (Austin, TX). Quality controlled, trimmed reads were assembled to a single contig at 27.3-fold coverage using Velvet version 1.2.10. The contig was confirmed to be complete by PCR using primers that face the upstream and downstream ends of the DNA. Products from the PCR amplification of the junctions of concatemeric molecules were sequenced by Sanger sequencing (Eton Bioscience, San Diego, CA). Genes were predicted using GeneMarkS (5) and corrected using software tools available on the Center for Phage Technology Galaxy instance (https://cpt.tamu.edu/galaxy-public/). Morphology was determined using transmission electron microscopy performed at the Texas A&M University Microscopy and Imaging Center.

Mushroom has an 87,709-bp genome with a G+C content of 39.03% and a coding density of 88.5%. Mushroom shares 48.6% nucleotide sequence identity with *Salmonella* phage Felix O1 (NC_005282) as determined by Emboss Stretcher (6). It is a member of the “Lytic 15” phage cluster as defined by Grose and Casjens (7). The G+C content was lower compared to the host (52.2%), a common feature of Felix O1-like phages (8, 9). Mushroom encodes twenty-three tRNAs, two of which are pseudo tRNA genes (10, 11). Interestingly, Mushroom does not encode the tmRNA, *ssrA*, encoded by Felix O1 (10). For annotation purposes, the genome has been opened to *rIIa*.

Mushroom encodes core genes representative of Felix O1-like phages involved in biosynthesis, replication, morphogenesis, and lysis (8, 12). Unlike the DNA polymerase of Felix O1, however, which is encoded by a single gene, the DNA polymerase of Mushroom exists as two genes whose products align with the single protein of Felix O1 (8). The region separating the two polymerase genes in Mushroom is 383-bp in length and has no obvious open reading frame corresponding to a homing endonuclease. The anaerobic ribonucleoside diphosphate reductase of Mushroom is disrupted by a free-standing homing endonuclease, as is seen in the ribonucleoside reductase of bacteriophage T4 (13). FelixO1, on the other hand has no reported T4-like intervening homing endonucleases (8). Mushroom encodes a T4-like lysis system with a soluble lysozyme, a class-III holin (1 transmembrane domain; N-in, C-out topology), and imbedded inner and outer membrane spanin proteins (14–16).

### Nucleotide sequence accession number.

The genome sequence of Mushroom was contributed as accession no. KP143762 to GenBank.
